# Ten-year atherosclerotic cardiovascular disease risk score in post-menopausal women with low bone mineral density

**DOI:** 10.1007/s40520-025-02957-1

**Published:** 2025-02-27

**Authors:** Kaiser Wani, Shaun Sabico, Nicola Veronese, Abeer A. Al-Masri, Nasser M. Al-Daghri

**Affiliations:** 1 Biochemistry Department, College of Science, King Saud University, Riyadh, 11451 Saudi Arabia; 2 Geriatrics and Internal Medicine, Saint Camillus International University of Health Sciences, Rome, 00131 Italy; 3 Department of Physiology, College of Medicine, King Saud University, Riyadh, 11451 Saudi Arabia

**Keywords:** Cardiovascular disease, ASCVD risk score, Lipids, HDL cholesterol, BMD, Post-menopausal women

## Abstract

**Background:**

Reports on the association between cardiovascular disease (CVD) risk and bone mineral density (BMD) remain inconsistent and hence more population-based studies on this subject are needed.

**Aims:**

This cross-sectional study aimed to evaluate the association between bone mineral density (BMD) at the lumbar spine (L1-L4) and femoral neck (right and left) with 10-year atherosclerotic cardiovascular disease (ASCVD) risk scores in Saudi postmenopausal women.

**Methods:**

A cohort of 1,450 postmenopausal women with risk factors for bone loss were analyzed using the data from the Chair for Biomarkers of Chronic Diseases (CBCD) Osteoporosis database. BMD at the lumbar spine and femoral neck was assessed using dual-energy X-ray absorptiometry (DXA). Anthropometric and biochemical parameters, including fasting glucose and lipid profiles, were measured. ASCVD risk scores were calculated using the ASCVD Risk Estimator Plus tool. BMD tertiles were analyzed for their association with ASCVD risk.

**Results:**

Women with osteoporosis had significantly lower BMI, waist and hip circumferences, and metabolic dysfunction markers compared to those with normal BMD. Significant negative correlations were observed between ASCVD risk scores and BMD at femoral neck sites in women with osteopenia and osteoporosis. Multivariate logistic regression indicated that women in the lowest BMD tertiles had significantly higher odds of intermediate to high ASCVD risk scores, with adjusted odds ratios of 1.90 for the lumbar spine, 2.19 for the right femoral neck, and 2.04 for the left femoral neck.

**Conclusions:**

The study identified significant associations between lower BMD at the lumbar spine and femoral neck sites and elevated 10-year ASCVD risk scores in postmenopausal women, particularly among those with osteopenia and osteoporosis. These findings demonstrate the importance of assessing cardiovascular risk in women with low BMD to enable early prevention and management strategies.

## Introduction

Cardiovascular disease (CVD) and osteoporosis are two significant health concerns affecting millions of people worldwide [1, 2]. CVD refers to a spectrum of conditions impacting the heart and blood vessels, including coronary artery disease, heart failure, and stroke [3, 4]. Osteoporosis, on the other hand, is characterized by reduced bone mass and microarchitectural deterioration of bone tissue, leading to increased fracture risk [5, 6]. Both conditions have been recognized as major public health burdens, particularly in aging populations [7]. Research has explored potential links between these conditions, specifically examining the association between cardiovascular disease risk and bone mineral density (BMD) [8, 9].

Cardiovascular diseases, including coronary artery disease and stroke, remain leading causes of mortality and morbidity worldwide and Saudi Arabia is no exception [10, 11]. According to the Saudi Ministry of Health, CVD accounts for a significant proportion of deaths in Saudi Arabia [12]. Identifying individuals at high risk of developing cardiovascular events is essential for implementing appropriate preventive measures and optimizing patient care. The ASCVD (Atherosclerotic Cardiovascular Disease) Risk Estimator Plus is a reliable risk assessment tool that combines multiple risk factors to estimate an individual’s 10-year risk of developing atherosclerotic cardiovascular disease [13]. The ASCVD score integrates various risk factors associated with cardiovascular disease into a comprehensive risk assessment. The key components considered in calculating the ASCVD score include age, sex, total cholesterol, high-density lipoprotein cholesterol (HDL-C), blood pressure, diabetes, smoking status, and antihypertensive treatment.

The ASCVD score is calculated using a validated algorithm which estimates the 10-year risk of experiencing a major cardiovascular event, including fatal and non-fatal myocardial infarction (heart attack) or stroke. Although many variants of the CVD risk assessment tool have been developed, the most recent guidelines from the American College of Cardiology / American Heart Association (ACC/AHA) [14, 15] recommends a tool called the ASCVD Risk Estimator Plus for risk estimate in people who do not already have CVD. This new score, like the older ones, is based on a collection of established risk factors to estimate the likelihood of an ASCVD event occurring within the next decade.

The association between cardiovascular risk and BMD has gained attention in recent years, and studying this relationship is important for several reasons. Osteoporosis, a condition characterized by low BMD and increased fracture risk, and CVD share common risk factors including advanced age, female sex, physical inactivity, smoking, and metabolic disorders. Understanding the interplay between these conditions can help identify shared mechanisms and potential interventions. Certain medications used to manage CVD, such as statins, beta-blockers, and anticoagulants, may influence BMD and bone health [16]. Studying the association between cardiovascular risk and BMD can help healthcare providers make informed decisions about medication use and evaluate their potential effects on bone health. Osteoporotic fractures can have severe consequences including increased disability, healthcare costs, and reduced quality of life especially in postmenopausal women [17–19].

Examining the link between cardiovascular risk and BMD can enable clinicians to identify individuals at heightened fracture risk and implement preventive strategies. Recent evidence suggests shared pathophysiological mechanisms between osteoporosis and CVD [20]. However, discrepancies in findings across studies may arise from differences in study populations, designs, confounding factors, and biological mechanisms. More population-based studies on this subject are needed to clarify these associations. In this context, the objective of this observational study was to evaluate the relationship between the 10-year ASCVD risk score and BMD at three bone sites (spine L1-L4, and right and left femoral-neck) in Saudi postmenopausal women.

## Materials and methods

The Osteoporosis Registry Database of the Chair for Biomarkers for Chronic Diseases (CBCD) at King Saud University (KSU) was employed for this cross-sectional study. This database contains clinical information on Saudi adults who completed bone mineral density (BMD) evaluations at several tertiary institutions in Riyadh, including King Fahad Medical City (KFMC), King Khalid University Hospital (KKUH), and King Salman Hospital (KSH), between 2013 and 2016 [21–23]. Participants were excluded from the study if they had a history of anti-osteoporotic treatment, were taking medications that influence bone metabolism, or had documented bilateral oophorectomy, hypogonadism, hypothyroidism, malignancies, hereditary bone disorders, or systemic diseases. The investigators strictly observed the applicable ethical standards with regard to the collection, storage, and analysis of biological samples. All participants received written informed consent prior to their enrollment in the study. The Institutional Review Board (IRB) of the College of Medicine at KSU in Riyadh, Saudi Arabia, granted ethical approval. This study was conducted in adherence to the STROBE guidelines for cross-sectional studies to ensure transparency and methodological rigor. A completed STROBE checklist has been provided as supplementary material (Table S1).

### Study design and participants

**Fig. 1:**
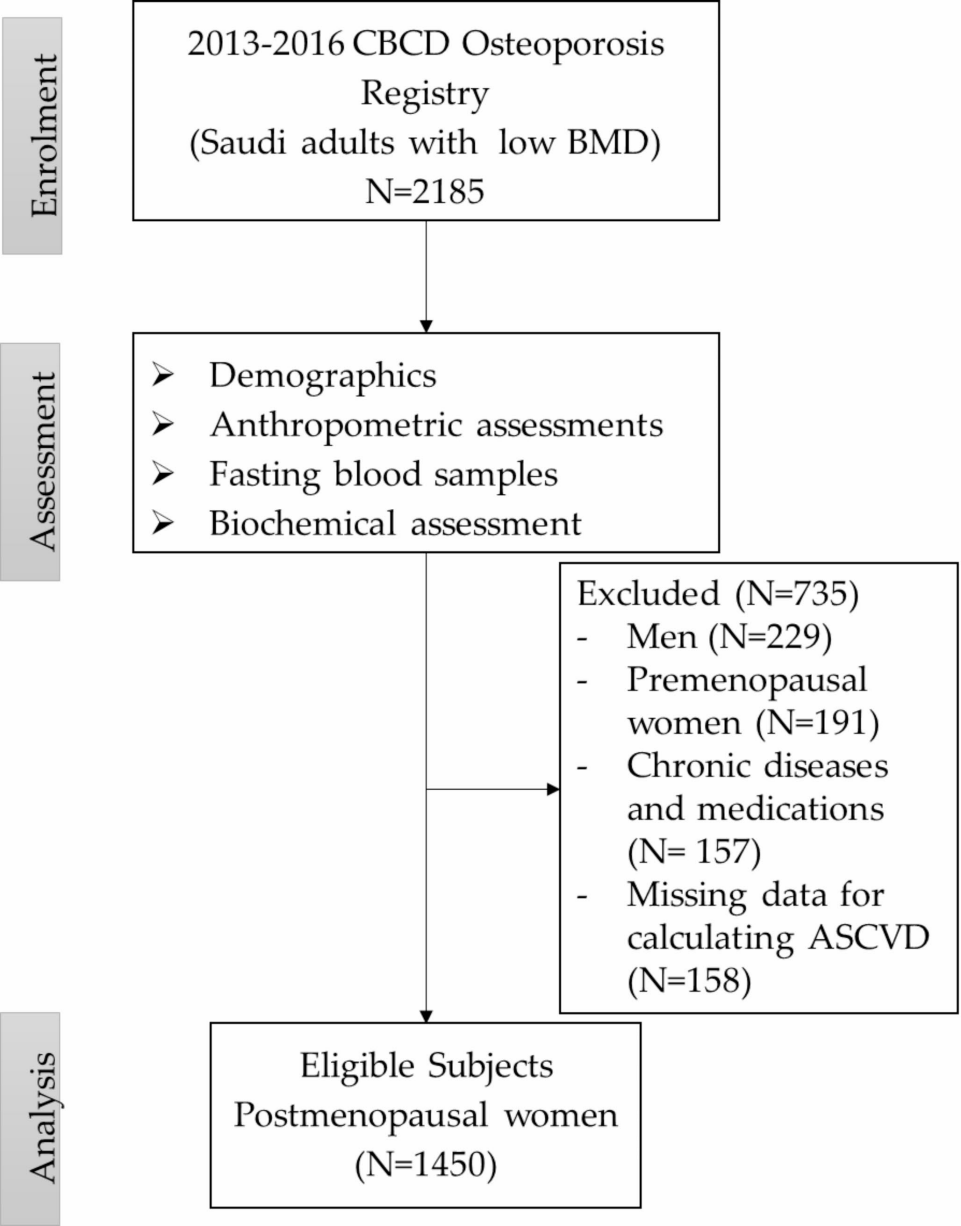
Flowchart of study participants

In this investigation, samples were obtained from a total of 1450 Saudi postmenopausal women from the Osteoporosis registry (*N* = 2185). Men were excluded, as were women who had not yet reached menopausal status at the time of recruitment, those with known malignancies and cardiovascular disorders, those who were taking medications that affected both BMD and ASCVD status, such as glucocorticoids, and those who lacked biochemical information that was required for the calculation of ASCVD. Demographic and clinical data, such as age, sex, medical history, and risk factors associated with bone loss (e.g., family history of osteoporosis, diabetes, or arthritis, thyroid disease, scoliosis of the spine, kyphosis, loss of height in the past two years, or a fracture in the past five years), was recorded. The flowchart of the study population is illustrated in Fig. 1.

### Bone mineral density (BMD) scan and the study groups

In all participants, bone mineral density (BMD) was assessed at three anatomical sites: the right femoral-neck, the left femoral-neck, and the lumbar vertebrae L1–L4, using dual-energy X-ray absorptiometry (DXA) equipment (Hologic Inc., Marlborough, MA, USA). The apparatus’ accuracy was verified using a standard phantom from the manufacturer, and the readings were obtained by a certified bone densitometry technician. The participants were stratified into three groups based on the T-scores calculated for BMD at the Spine L1-L4 bone site: the normal BMD group (T-score of -1.0 and above), the osteopenia group (T-score < -1.0 and >-2.5), and the osteoporosis group (T-score < -2.5). The cut-offs utilized in this study were derived from the World Health Organization [24] and employed by the national and regional guidelines to identify individuals at risk for bone loss [25–27].

### Sample collection, anthropometric and biochemical evaluations

A The registry was used to retrieve information on the anthropometric measurements (body mass index, BMI; waist and hip circumference; and blood pressure) and biochemical parameters (glucose and lipid profile) of the included participants. In summary, the height (cm) and weight (kg) of the participants were measured using a standardized scale that was supplied with an affixed stadiometer. The body mass index (BMI) was calculated. The standard non-elastic tape measure was employed to measure the circumferences of the waist (cm) and hips (cm). Systolic and diastolic blood pressure (mmHg) were measured twice with a 15-minute interval in a seated position using an automated instrument (Omron-705CP; Omron Corp., Tokyo, Japan). In the CBCD laboratory at KSU, Riyadh, Saudi Arabia, all fasting blood samples were evaluated and stored. Fasting glucose, total cholesterol, HDL-cholesterol, triglycerides, calcium, and albumin were routinely measured using a biochemistry analyzer (Konelab 20XT, Thermo Scientific, Vantaa, Finland) [28], as was previously reported. The Luminex multiplex platform (Luminexcorp, Texas) was employed to assess the circulating levels of insulin (kit Id: HBNMAG-51 K).

### ASCVD calculation

The ASCVD Risk Estimator Plus, created by the American College of Cardiology and the American Heart Association (ACC/AHA), is an evidence-based digital tool that evaluates the likelihood of Atherosclerotic Cardiovascular Disease (ASCVD) events occurring in the next ten years in individuals who do not currently have cardiovascular disease (CVD). This cutting-edge program utilizes a comprehensive array of validated risk elements to produce estimates. The risk factors that are considered include age, gender, systolic and diastolic blood pressures, levels of total cholesterol, HDL cholesterol, and LDL cholesterol in the blood, as well as factors related to race, history of diabetes, smoking habits, and the use of statins, hypertension treatment, or aspirin [29]. In order to calculate the ASCVD risk score, the data of each participant namely the risk factors listed above were methodically entered into the ASCVD Risk Estimator Plus. Afterwards, the program calculated a 10-year ASCVD risk score for each participant. The scores obtained were further classified into risk categories: low risk (< 7.5%), intermediate risk (7.5–19.9%), and high risk (≥ 20%).

### Statistical analysis

We conducted the sample size determination using G*power 3.1 statistical software. Based on an expected effect size of 0.25 for differences in cardiovascular risk among the three groups derived from prior literature, a significance level of 0.05, and a power of 0.90, a minimum sample size of 145 per group was required. The final sample size used in this study was sufficient to perform robust statistical analyses, including subgroup comparisons and adjustments for confounding factors. We used the SPSS version 28.0 (SPSS, Inc., Chicago, IL, USA) to analyze the data presented in this study. The participants were divided into three groups: normal BMD, osteopenia and osteoporosis as per the cut-offs given above. Continuous variables following a Gaussian distribution were presented as mean ± standard deviation (SD), while non-Gaussian variables were described as median and interquartile range (IQR). The frequencies and the associated percentages in the group was utilized to present the categorical variables. Differences between these groups were compared using the one-way analysis of variance (ANOVA), and the chi-square test. A bivariate correlation analysis was conducted to check the association of the 10-year ASCVD risk scores with other measured parameters in the study, the test employed was Spearman’s test and the data was presented as correlation coefficient and associated p-values. The participants were also divided into tertiles based on the BMD’s at the three bone sites with tertile 1 representing the lowest BMD and tertile 3 representing the highest BMD. Also, the ASCVD risk scores were categorized as low, and intermediate to high risk groups based on the cut-offs given above. A multivariate logistic regression analysis was performed using the categorical variables of BMD tertiles and ASCVD risk categories. The odds ratio of having intermediate to high ASCVD risk scores in lower BMD tertiles, compared to the highest tertile, was calculated. The analysis was adjusted for age, BMI, and additional risk factors for low BMD, including a family history of diabetes, osteoporosis, and arthritis, as well as conditions such as scoliosis of the spine, kyphosis, and recent loss of height. The study yielded data in the form of odds ratio (OR) with corresponding 95% confidence interval. The threshold for statistical significance was defined as *p* < 0.05. The figures were prepared using MS Excel 2010.

## Results

### Characteristics of the study subjects

A total of 1450 post-menopausal Saudi women with a mean age of 56.81 years were recruited in this study. T-scores at the spine L1-L4 bone sites were used to categorize the subjects into normal (*N* = 439), osteopenia (*N* = 630), and osteoporosis (*N* = 381) groups. The general characteristics of the subjects in the study groups are presented in Table 1. The subjects in the osteoporosis group were older, and consequently had higher average post-menopausal years; however, they had significantly lower BMI, lower waist and hip circumferences, and lower fasting glucose, insulin, and triglyceride levels compared to the normal BMD group. The average BMD in g/cm2 at three bone sites- spine, right femoral-neck, and left femoral-neck in the three study groups is presented in the table.

**Table 1:** Characteristics of the study subjects

	All (1450)	Normal (439)	Osteopenia (630)	Osteoporosis (381)	*p*
T- score Spine L1-L4	-1.7 (-2.5, -0.8)	-0.2 (-0.6,0.5)	-1.8 (-2.1, -1.4)	-2.9 (-3.3, -2.7)	< 0.001
BMD (Spine) g/cm^2^	0.97 ± 0.2	1.17 ± 0.1	0.96 ± 0.1	0.82 ± 0.1	< 0.001
BMD (FN-right) g/cm^2^	0.92 ± 0.2	1.02 ± 0.1	0.91 ± 0.1	0.82 ± 0.2	< 0.001
BMD (FN-Left) g/cm^2^	0.92 ± 0.2	1.03 ± 0.1	0.91 ± 0.1	0.81 ± 0.1	< 0.001
**Clinical and Biochemical characteristics**					
Age	56.81 ± 4.7	55.19 ± 4.6	56.85 ± 4.6	58.61 ± 4.4	< 0.001
Age at Menarche	13.17 ± 1.5	12.98 ± 1.4	13.16 ± 1.5	13.42 ± 1.5	< 0.001
Years-post menopause	6 (3,13)	4 (3,8)	6 (3,12)	10 (5,15)	< 0.001
BMI	32.94 ± 6.2	34.31 ± 6.1	33.34 ± 5.9	30.69 ± 6.0	< 0.001
Waist circumference	99.81 ± 14.4	102.61 ± 13.9	100.10 ± 13.3	96.10 ± 15.7	< 0.001
Hip circumference	108.80 ± 13.5	111.70 ± 12.7	109.50 ± 12.3	104.62 ± 15.2	< 0.001
Systolic-BP	126.22 ± 17.8	126.22 ± 17.2	126.46 ± 17.6	125.83 ± 18.7	0.861
Fasting glucose (mmol/l)	6.56 (5.4,9.5)	7.00 (5.5,9.7)	6.54 (5.4,9.8)	6.25 (5.3,8.5)	0.031
Insulin (µU/ml)	10.7 (4.6,16.8)	8.92 (4.3,15.2)	13.24 (8.8,23)	7.12 (3.1,16.1)	< 0.001
CHOL (mg/dl)	197.07 ± 40.6	197.48 ± 41	198.46 ± 40.6	194.31 ± 40.1	0.281
HDL-Chol (mg/dl)	44.67 ± 15.4	43.52 ± 14.8	45 ± 15.3	45.44 ± 16.4	0.161
Triglyceride (mmol/l)	1.54 (1.2,2.1)	1.60 (1.2,2.3)	1.56 (1.2,2.1)	1.46 (1.1,2)	0.006
Albumin (g/L)	37.82 ± 5.5	37.77 ± 5.4	38.06 ± 5.5	37.5 ± 5.6	0.413
Calcium (mmol/L)	2.34 ± 0.3	2.33 ± 0.3	2.33 ± 0.3	2.35 ± 0.3	0.373

Note: Data is presented as mean ± standard deviation and median (quartile1, quartile3) for Gaussian and non-gaussian continuous variables respectively. BMD is ‘Bone Mineral Density’, FN is ‘Femoral-neck’, CHOL is ‘Total cholesterol’, and HDL-Chol is High-density Lipoprotein Cholesterol. The difference between the study groups was calculated using the ANOVA and is represented by the p-value. The non-gaussian variables were log-transformed before tests were applied. *P* < 0.05 was taken as significant

### Risk factors of low BMD and cardiovascular diseases

The prevalence of risk factors for low BMD and cardiovascular diseases in the study groups is presented in Table 2. The osteoporosis group had the highest proportion of subjects over 50 years of age (*p* < 0.001). The proportion of subjects with scoliosis of the spine, kyphosis, and those who lost height in the last 2 years was highest in the osteoporosis group. The proportion of subjects with high cholesterol, low HDL-cholesterol, and hypertension was comparable in all three groups. Those having the smoking history and ones with high cholesterol levels was higher in osteopenia and osteoporosis groups compared to the normal group; however, diabetes was less prevalent in the osteoporosis group compared to the normal group. The proportion of subjects with low 10-year ASCVD risk scores was highest in the normal group while ones with high ASCVD risk score was highest in the osteoporosis group (*p* < 0.001). The mean 10-year ASCVD risk score also increased from normal (3.94) to osteopenia (4.91) groups and was highest in the osteoporosis (6.11) group (*p* < 0.001).

**Table 2:** Risk factors of low BMD and cardiovascular diseases in the study groups

	All (1450)	Normal (439)	Osteopenia (630)	Osteoporosis (381)	*p*
**Low BMD risk factors**					
Age > 50 years	1315 (90.7)	375 (85.4)	577 (91.6)	363 (95.3)	< 0.001
Vitamin D Deficiency	294 (32.5)	110 (37.3)	122 (31.1)	62 (28.6)	0.084
Family History-Diabetes	795 (54.8)	289 (65.8)	339 (53.8)	167 (43.8)	< 0.001
Family History-Osteoporosis	101 (7.0)	41 (9.3)	33 (5.2)	27 (7.1)	0.035
Family History-Arthritis	81 (5.6)	34 (7.7)	29 (4.6)	18 (4.7)	0.062
Thyroid Disorder	106 (7.8)	38 (8.9)	42 (7.0)	26 (7.6)	0.539
Rheumatoid Arthritis	145 (10.6)	52 (12.2)	54 (9.1)	39 (11.4)	0.237
Barium test-last two weeks	133 (9.7)	44 (10.3)	50 (8.3)	39 (11.4)	0.278
Scoliosis of Spine	110 (8.0)	29 (6.8)	40 (6.7)	41 (12.0)	0.009
Kyphosis	48 (3.5)	12 (2.8)	12 (2.0)	24 (7.0)	< 0.001
Lost height-last 2 years	127 (9.3)	35 (8.2)	43 (7.2)	49 (14.3)	< 0.001
Fracture-last 5 years	129 (9.4)	32 (7.5)	57 (9.5)	40 (11.7)	0.146
**Cardiovascular risk factors**					
High Cholesterol	794 (54.8)	239 (54.4)	332 (52.7)	223 (58.5)	0.194
High Triglycerides	856 (59.2)	241 (54.9)	369 (58.9)	246 (64.7)	0.016
Low HDL-C	459 (31.7)	124 (28.2)	208 (33.0)	127 (33.3)	0.183
Treatment-BP	363 (25)	79 (17.9)	156 (24.8)	88 (23.1)	0.408
Diabetes	900 (62.1)	295 (67.2)	388 (61.6)	217 (57.0)	0.011
Smoking History	331 (22.8)	84 (19.1)	159 (25.2)	88 (23.1)	< 0.001
**10-year ASCVD**					
10-year ASCVD score	4.78 (2.3,11.2)	3.94 (2.1,7.2)	4.91 (2.5,13.4)	6.11 (2.7,15.4)	< 0.001
Low	945 (65.2)	341 (77.7)	388 (61.6)	216 (56.7)	< 0.001
Intermediate	319 (22)	79 (18.0)	143 (22.7)	97 (25.5)	
High	186 (12.8)	19 (4.3)	99 (15.7)	68 (17.8)	

**Note**: Data is presented as N (%) and median (quartile1, quartile3) for categorical and non-gaussian continuous variables respectively. The difference between the study groups was calculated using the Chi-square test and is represented by the p-value. *P* < 0.05 was taken as significant.

**Fig. 2:**
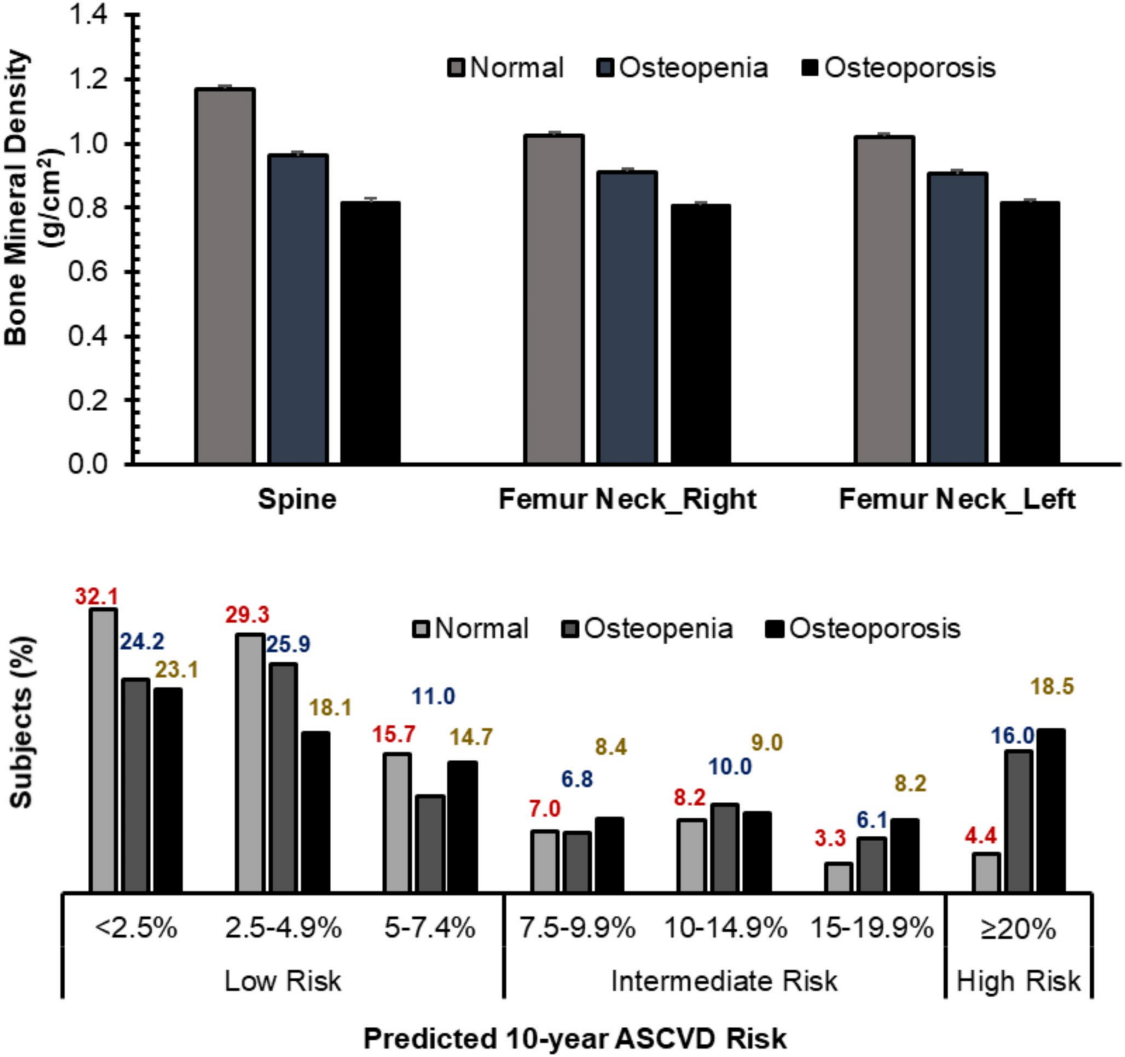
Average BMD at three bone sites, and the percentage of subjects with 10-year ASCVD risk categories in the three study groups

The average BMD at the three bone sites and the proportion of subjects with 10-year ASCVD risk score categories in study subjects with normal, osteopenia, and osteoporotic BMD is presented as bar graphs in Fig. 2.

### Bivariate correlation between 10-year ASCVD risk score and other measured parameters in the study groups

A bivariate correlation analysis was done between the 10-year ASCVD score and other measured parameters in the three study groups and the results were presented in Table 3. As expected, a significant positive correlation was seen with age and BMI in all three groups (*p* < 0.001 in all). The ASCVD risk score also showed significant positive correlations with waist circumference, systolic and diastolic BP, fasting glucose, and triglyceride levels (*p* < 0.001 in all) and a significant negative correlation with HDL-Cholesterol levels (*p* < 0.001) in all three study groups. The bivariate association of ASCVD risk score with BMD suggested a negative correlation at right and left femoral-neck bone sites in osteopenia (*r* = -0.12, *p* = 0.008 and *r*= -0.15, *p* < 0.001 respectively) and osteoporosis (*r* = -0.14, *p* = 0.011 and *r*= -0.21, *p* < 0.001 respectively) groups only and not in the normal BMD group.

**Table 3:** Bivariate correlations between 10-year ASCVD risk score and other parameters

	All (1450)		Normal (439)		Osteopenia (630)		Osteoporosis (381)	
Parameters	R	*p*	R	*p*	R	*p*	R	*p*
**Clinical**								
Age	0.65	< 0.001	0.69	< 0.001	0.58	< 0.001	0.69	< 0.001
Age at Menarche	0.01	0.97	0.08	0.118	-0.04	0.34	-0.06	0.268
Years-post menopause	0.43	< 0.001	0.43	< 0.001	0.38	< 0.001	0.44	< 0.001
BMI	0.08	0.003	0.11	0.021	0.12	0.002	0.11	0.028
Waist circumference	0.21	< 0.001	0.31	< 0.001	0.25	< 0.001	0.28	< 0.001
Hip circumference	0.03	0.299	0.06	0.227	0.06	0.15	0.07	0.195
Systolic-BP	0.65	< 0.001	0.55	< 0.001	0.68	< 0.001	0.73	< 0.001
Diastolic-BP	0.28	< 0.001	0.22	< 0.001	0.34	< 0.001	0.29	< 0.001
**Biochemical**								
Fasting glucose	0.32	< 0.001	0.32	< 0.001	0.37	< 0.001	0.31	< 0.001
Insulin	0.07	0.168	-0.01	0.895	0.08	0.385	0.05	0.583
Cholesterol	-0.01	0.855	0.07	0.165	-0.03	0.409	-0.03	0.532
HDL-Chol	-0.28	< 0.001	-0.32	< 0.001	-0.28	< 0.001	-0.26	< 0.001
Triglyceride	0.21	< 0.001	0.31	< 0.001	0.21	< 0.001	0.18	< 0.001
Albumin	-0.07	0.028	-0.06	0.265	-0.04	0.386	-0.12	0.054
Calcium	-0.02	0.563	0.02	0.721	-0.07	0.171	0.01	0.976
**BMD**								
BMD (Spine)	-0.11	0.003	0.10	0.052	0.01	0.896	-0.09	0.078
BMD (FN-Right)	-0.17	< 0.001	-0.02	0.714	-0.12	0.008	-0.14	0.011
BMD (FN-Left)	-0.21	< 0.001	-0.06	0.303	-0.15	< 0.001	-0.21	< 0.001

**Note**: Data is presented as correlation coefficient and the associated p-values. BMD is ‘Bone Mineral Density’, FN is ‘Femoral-neck’, CHOL is ‘Total cholesterol’, and HDL-Chol is High-density Lipoprotein Cholesterol. *P* < 0.05 was taken as significant.

**Fig. 3:**
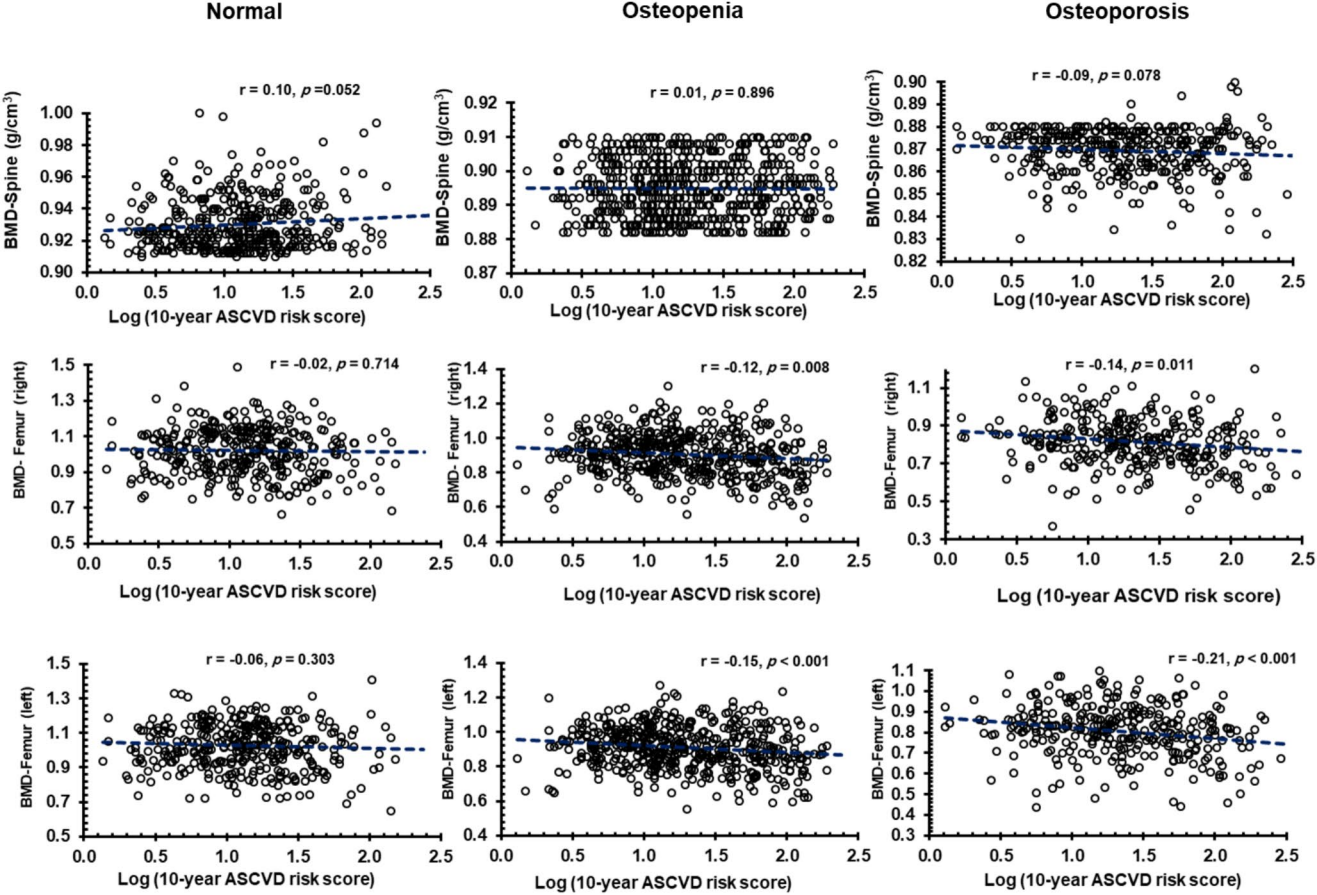
Scatterplots representing the bivariate correlation of the BMD at three bone sites with the 10-year ASCVD risk scores

The bivariate correlations between the 10-year ASCVD risk scores and BMD values at the three bone sites is plotted as scatter plots in Fig. 3.

### Association between BMD and 10-year ASCVD risk scores

The 10-year ASCVD risk scores were categorized as low-risk, and intermediate to high-risk levels and the BMD values at the three bone sites were divided into tertiles. The resultant categorical variables were used to conduct a multivariate logistic regression analysis and the odds of having intermediate to high ASCVD risk scores in lower tertiles of BMD compared to the highest tertile were calculated and adjusted in models ‘a’, ‘b’, and ‘c’ respectively with age, +BMI, and + risk factors of low BMD including a family history of diabetes, osteoporosis, and arthritis, and whether having conditions like scoliosis of the spine, kyphosis and lost height in last 2 years. The results are presented in Table 4. The analysis suggests that the lowest tertile of BMD had higher odds of intermediate to high ASCVD risk scores compared to the highest tertile (tertile 3) at all three bone sites even after multiple adjustments (Odds ratio and 95% confidence intervals of 1.90 (1.2–2.9), *p* = 0.004; 2.19 (1.3–3.6), *p* = 0.002, and 2.04 (1.2–3.4), *p* = 0.006 at spine (L1-L4), femoral-neck (right) and femoral-neck (left) bone sites respectively) indicating an independent significantly negative association between BMD and ASCVD risk scores. Also, the odds of having intermediate to high ASCVD risk scores for osteopenia and osteoporosis groups were significantly higher even after multiple adjustments (OR = 2.06, 95% CI = 1.3–3.3, *p* = 0.003; and OR = 2.01, 95% CI = 1.5 -3.0, *p* < 0.001) when compared to the normal BMD group.

**Table 4:** Multivariate logistic regression analysis for association between BMD and 10-year ASCVD risk scores

BMD	10-year ASCVD score			Regression analysis					
	Tertiles	Low Risk	Intermediate to High Risk	Models	Tertile 1		Tertile 2		Tertile 3
					OR, 95% CI	*p*	OR, 95% CI	*p*	
BMD at L1-L4 Spine	Tertile 1	58.9	41.1	Univariate	2.07 (1.6–2.7)	< 0.001	1.90 (1.4–2.5)	< 0.001	Ref.
	Tertile 2	60.9	39.1	a	1.38 (1.0 -1.9)	0.033	1.60 (1.2–2.1)	0.002	Ref.
	Tertile 3	74.8	25.2	b	1.53 (1.1–2.1)	0.007	1.66 (1.2–2.2)	< 0.001	Ref.
				c	1.90 (1.2–2.9)	0.004	2.26 (1.5–3.4)	< 0.001	Ref.
BMD Femoral-neck (right)	Tertile 1	50.9	49.1	Univariate	2.49 (1.8–3.3)	< 0.001	1.32 (1.0 -1.8)	0.071	Ref.
	Tertile 2	66.1	33.9	a	1.61 (1.2–2.2)	0.004	1.05 (0.8–1.5)	0.775	Ref.
	Tertile 3	72.1	27.9	b	1.96 (1.4–2.8)	< 0.001	1.21 (0.9–1.7)	0.269	Ref.
				c	2.19 (1.3–3.6)	0.002	1.10 (0.7–1.8)	0.698	Ref.
BMD at Femoral-neck (left)	Tertile 1	51.4	48.6	Univariate	2.63 (1.9–3.5)	< 0.001	1.56 (1.1–2.1)	0.004	Ref.
	Tertile 2	64.1	35.9	a	1.78 (1.3–2.4)	< 0.001	1.18 (0.9–1.6)	0.313	Ref.
	Tertile 3	73.5	26.5	b	2.25 (1.6–3.2)	< 0.001	1.39 (1.0–2.0)	0.051	Ref.
				c	2.04 (1.2–3.4)	0.006	1.23 (0.8 -2.0)	0.401	Ref.
**Diagnosis**		**10-year ASCVD score**		**Regression analysis**					
		**Low Risk**	**Intermediate to** ** High Risk**	**Models**	**Osteoporosis**		**Osteopenia**		**Normal**
					**OR**,** 95% CI**	***p***	**OR**,** 95% CI**	***p***	
Normal		77.1	22.9	Univariate	2.66 (2.0 -3.6)	< 0.001	2.17 (1.6–2.9)	< 0.001	Ref.
Osteopenia		61.1	38.9	a	1.63 (1.2–2.3)	0.004	1.69 (1.3–2.3)	< 0.001	Ref.
Osteoporosis		55.9	44.1	b	1.83 (1.3–2.6)	< 0.001	1.78 (1.4–2.5)	< 0.001	Ref.
				c	2.06 (1.3–3.3)	0.003	2.01 (1.5 -3.0)	< 0.001	Ref.

**Note**: Data is presented as odds ratio, 95% confidence intervals, and associated p-values. *P* < 0.05 was taken as significant.

## Discussion

The current study investigated the association between BMD and ASCVD risk in Saudi postmenopausal women. The multivariable logistic regression analysis performed in this study suggested that post-menopausal women in the lowest tertile of BMD had significantly higher odds of having intermediate to high ASCVD risk scores across the three studied bone sites, even after adjusting for age, BMI, and other risk factors. The data also indicated that post-menopausal women with osteoporosis had the highest ASCVD risk scores compared to those with normal BMD or osteopenia. Conclusively, our findings suggest a potential link between lower BMD and increased ASCVD risk, but the strength of this association limits the extrapolation of these findings to clinical practice. The results of this cross-sectional observational study however highlight the importance of considering cardiovascular risk assessments in postmenopausal women with low BMD, as this population may be at an elevated risk for both osteoporosis and cardiovascular events. These findings signify the need for comprehensive management approaches that address both bone health and cardiovascular disease prevention in postmenopausal women.

To our knowledge, this is the first study to investigate the relationship between BMD and the calculated ASCVD risk scores. Some of the earlier studies on the subject focused on the association between BMD and actual cardiovascular events. Despite this difference, our findings align with some earlier studies that have reported an inverse relationship between BMD and cardiovascular risk factors. For instance, Tankó et al. [30] identified an association between reduced BMD and greater cardiovascular risk, which is consistent with our finding that lower BMD at the femoral neck and spine is linked to higher ASCVD risk scores. Similarly, Ye et al. [31] reported that reduced BMD was related to greater cardiovascular risk in Chinese postmenopausal women, supporting our findings in a different population. In alignment with our observations, although employing alternative cardiovascular risk measures, Barbour et al. [32] also observed an inverse relationship between BMD and cardiovascular disease in older women.

Multiple studies in the literature suggest that the link between BMD and cardiovascular risk may not be a simple inverse correlation. In some studies, the inverse association lost significance after adjustment with confounders [33] indicating the complexity of this association and the influence of additional confounders. Studies like the ones by Bhatta et al. [34] found no evidence of an association between BMD and cardiovascular events in 22,857 adults from The Trøndelag Health Study in Norway suggesting that the relationship between bone health and cardiovascular risk may not be consistent across different populations. Some other studies [35] reported links between higher BMD and coronary artery calcification pointing to a potential increase in CVD risk among women with higher bone mass.

The biological mechanisms connecting BMD and CVD risk in postmenopausal women are not yet completely understood; nevertheless, inflammation and shared risk factors such as aging and hormonal changes have been suggested as possible common pathways [36–38]. These findings correspond with prior research revealing a correlation between decreased bone mineral density and heightened cardiovascular risk, perhaps suggesting that osteoporosis and cardiovascular disease share similar pathophysiological mechanisms including the influence of factors like age, lifestyle choices, and metabolic changes linked to menopause [39–41]. The divergent findings about the relationship between BMD and cardiovascular disease may be ascribed to multiple differences between studies like age of participants, gender, and ethnicity, since various groups may demonstrate unique associations between BMD and CVD and also variations in research design, methodology, and sample sizes [42, 43]. Moreover, the complex interplay of underlying biological processes, including the interaction between bone metabolism and vascular calcification, may affect these associations across different studies.

The inverse association between BMD and ASCVD risk in postmenopausal women, found in this study, may be explained by several interconnected biological mechanisms. Chronic low-grade inflammation is a well-recognized contributor to both low BMD and high CVD risk. Inflammation promotes osteoclast activation and bone resorption, leading to decreased BMD [36, 44]. Simultaneously, inflammatory markers like C-reactive protein and interleukin-6 accelerate atherosclerosis by increasing endothelial dysfunction, lipid deposition, and vascular calcification [45, 46]. Inflammation levels thereby create an environment conducive to bone loss and vascular plaque accumulation, linking the decline in bone health with ASCVD progression. Studies have shown that anti-inflammatory treatments can modestly improve BMD and reduce cardiovascular risk, suggesting that inflammation control could be pivotal in mitigating the dual impact of osteoporosis and ASCVD [47, 48].

Low BMD and ASCVD risk are significantly influenced by shared risk factors, including age, smoking, sedentary behavior, and poor nutrition. Advancing age is associated with a natural decline in BMD, exacerbated by lifestyle factors like smoking and physical inactivity, which also elevate ASCVD risk [49, 50]. Physical inactivity impacts both bone and cardiovascular health, as exercise supports BMD maintenance and improves vascular function [51]. This overlap of lifestyle risk factors explains the simultaneous increase in osteoporosis and CVD risk in older populations [52]. Furthermore, estrogen plays a critical role in bone health by inhibiting bone resorption and its protective role extends to cardiovascular health by supporting vascular integrity [53]. Post-menopause, the sharp decline in estrogen levels leads to accelerated bone loss and heightened CVD risk due to an increase in both osteoclast activity in bones and inflammatory and lipid profiles that promote atherosclerosis which may leave these women more vulnerable to ASCVD risk, creating a dual burden post-menopause.

Cardiovascular risk assessment in postmenopausal women with low BMD is increasingly important due to the shared pathways between osteoporosis and CVD. Evidence, including the findings of this study, as discussed above, suggests that low BMD may also signal elevated ASCVD risk, which further highlights the potential for BMD as a marker for cardiovascular health assessment in this population. Consequently, the integration of cardiovascular risk assessments in postmenopausal women with low BMD assessments could provide valuable insights and enhance early intervention opportunities for CVD. A more holistic screening approach in clinical settings and a dual assessment strategy for osteoporosis and cardiovascular risk in postmenopausal women may be highly beneficial [54, 55]. Screening for ASCVD in women with low BMD could allow for the early detection of subclinical cardiovascular issues, particularly in women who might otherwise be classified as low risk based on traditional CVD risk factors alone. Incorporating regular cardiovascular screenings into osteoporosis care protocols would also help capture and monitor patients at risk for CVD complications [56]. Additionally, the study’s findings could influence preventive strategies by promoting lifestyle and pharmacologic interventions that address both conditions [57]. Recognition of the shared risk factors, such as inflammation, sedentary lifestyle, and smoking, etc., may help clinicians to implement comprehensive lifestyle modifications tailored to improve both bone and cardiovascular health. Nutritional guidance emphasizing calcium, vitamin D, and heart-healthy nutrients could be prioritized as prevention strategies for this at-risk population [58]. Besides, the public health implications of this dual-risk profile assessment may be significant as it could improve early intervention rates, lower healthcare costs, and enhance the quality of life among aging females especially in this population with a high prevalence of osteoporosis and CVD among postmenopausal women [59, 60].

Our study has several limitations that must be considered when interpreting the findings. First, the cross-sectional design prevents us from establishing causal relationships between BMD and ASCVD risk. While the observed correlations are statistically significant, their strength is relatively weak, which limits the direct clinical applicability of these results. These associations, therefore, should be interpreted with caution, as they may not fully capture the complex interplay of factors contributing to both osteoporosis and cardiovascular risk. Second, the study population was drawn from tertiary hospitals and may vary in health statuses or lifestyle factors when compared to the general post-menopausal population and hence may introduce selection bias and limit the generalizability of the findings to broader populations. A possibility of information bias in health data captured in this study such as menopausal status and years past menopause, etc. maybe there as such data was based on self-reported questionnaire information.

Additionally, we did not account for certain confounders, such as detailed dietary intake, physical activity levels, or inflammatory markers, which may mediate or confound the observed relationship. Despite these limitations, the study possesses several strengths, particularly its substantial sample size and thorough data collection from various tertiary hospitals, which strengthen the reliability and generalizability of the findings. Moreover, we employed the ASCVD Risk Estimator Plus, a validated and commonly used tool for calculating cardiovascular risk, in this study enhancing the precision of cardiovascular risk evaluations. Future research should focus on longitudinal studies to determine the causal relationship between BMD and cardiovascular risk, as well as intervention studies to investigate whether enhancing BMD could have a beneficial effect on cardiovascular outcomes. Future longitudinal studies are needed to explore the causal relationship between BMD and ASCVD risk. Additionally, interventional studies examining whether improving BMD through lifestyle or pharmacologic measures influences cardiovascular outcomes would provide valuable insights into the clinical implications of our findings.

## Conclusion

This study highlighted a statistically significant inverse relationship between BMD and ASCVD risk in Saudi postmenopausal women, suggesting a potential interplay between bone health and cardiovascular risk factors. The limited strength of the observed associations and the cross-sectional nature of the study however restrict its clinical applicability. While these findings contribute to the growing body of literature exploring the connection between osteoporosis and cardiovascular health, they should primarily be viewed as hypothesis-generating rather than conclusive evidence. These findings though emphasize the significance of monitoring cardiovascular risk in postmenopausal women who have low BMD. This method could improve the early detection and preventive treatment of osteoporosis and cardiovascular disease. Future longitudinal studies with larger sample sizes are required to further explore this relationship and determine its potential implications for clinical practice and preventive strategies.

## Data Availability

Data is provided within the manuscript or supplementary information files.
